# Supporting international medical graduates–what can be done better? A sequential explanatory mixed-methods study

**DOI:** 10.1371/journal.pone.0330558

**Published:** 2025-08-19

**Authors:** Sunita Joann Rebecca Healey, Kristy Fakes, Bunmi S. Malau-Aduli, Balakrishnan R. Nair

**Affiliations:** 1 School of Medicine and Public Health, College of Health, Medicine and Wellbeing, The University of Newcastle, Callaghan, New South Wales, Australia; 2 Equity in Health and Wellbeing Research Program, Hunter Medical Research Institute, New Lambton Heights, New South Wales, Australia; 3 Centre for Medical Professional Development, Hunter New England Local Health District, New Lambton Heights, New South Wales, Australia; 4 Heart and Stroke Research Program, Hunter Medical Research Institute, New Lambton Heights, New South Wales, Australia; 5 College of Medicine and Dentistry, James Cook University, Townsville, Queensland, Australia; King Saud bin Abdulaziz University for Health Sciences, SAUDI ARABIA

## Abstract

**Background:**

International medical graduates (IMGs) are a vital health workforce globally. Several challenges specific to IMGs warrant institutions to make necessary implementations to support their transition to working in host countries. To date, recommendations to support IMGs are frequently postulated based on challenges identified in the literature. However, there is a scarcity of information regarding IMGs’ perceptions of these supports, and how supports can be improved, from an IMG perspective.

**Methods:**

A sequential explanatory mixed methods study was conducted to deeply understand how current and future supports might enhance the IMG experience. Between October and December 2023, a cross-section of IMGs across Australia were surveyed, recruited through a variety of non-random avenues. Following this, a qualitative phase comprised of interviewing participants. Findings are reported in line with Good Reporting of a Mixed Methods Study recommendations.

**Results:**

In total, 192 survey participants and 36 interviewees completed the relevant ‘supports’ section of the survey and semi-structured interview respectively. By collating the results of both the survey and interviews, findings were organized into the following categories: 1) Supports experienced by IMGs in Australia- 1a) Formalised institutional or workplace support, 1b) Informal networks and community support; 2) How institutions can better support IMGs- 2a) Improve operation of bureaucratic systems, 2b) Institutions can better mobilise IMGs by supporting integration and skill development, 2c) IMGs call for changes to reduce discrimination and exploitation risks; 3) How IMGs can better support themselves- 3a) Attitude and adaptation are important, 3b) Be prepared and seek advice early, 3c) Consider life stages.

**Conclusions:**

IMGs identified a range of useful current and recommended supports in and out of the workplace. In addition, IMGs have a role to play in harnessing their own agency for enhancing their own experiences in host countries. Further research and policy attention is required to ensure that institutions provide fair working conditions, support integration and prioritise cultural safety in the workplace.

## Introduction

Several developed countries rely on the movement of international medical graduates (IMGs) to their shores to fill critical workforce shortages in areas of need and to service vulnerable populations [1–3]. As developed countries like the UK, USA, Canada and Australia work on long-term measures to increase their local supply of doctors, these countries recognise that the recruitment of IMGs remains a priority for workforce bolstering in the short and medium term [2–5].

Several factors have been identified as driving the choices made by doctors to migrate internationally, such as limited career options and socio-political instability in their homeland or better quality of life and income in host countries [6]. Given their importance to the security of a nation’s health, it is not surprising that there is growing interest in how institutions can best support IMGs in transitioning to host countries. Motala’s 2019 scoping review into experiences of IMGs and factors facilitating adjustment found a relative paucity of studies exploring IMG supports [7]. Several researchers recognise the importance of supporting IMGs in the workplace, and thereby postulate recommendations based on challenges identified in studies. Examples include programs targeting preparedness for practice, communication skills, experience sharing, review of institutional structures and promoting cultural safety in the workplace [8–12]. Such supports have been suggested in response to identified challenges of integration, perceived inequity, differences in language and culture, and migration stress [10–12]. A few evaluations of IMG projects are published in the literature, tending to focus on medical education, training and transition [13–16]. There remains a dearth in understanding how IMGs perceive the utility of commonly proposed supports. Despite consumer consultation being a widely recognised factor for instrumenting successful change, there is a relative lack of evidence demonstrating consultation of IMGs for input and development of ideas exploring options for support.

Therefore, in this study we consulted a sample of IMGs across Australia to ask about supports which have been proposed in the literature. By undertaking a mixed-methods study, we sought to deeply understand how IMGs experienced and viewed the supports which they had already received, both in and outside the workplace. Also, we were interested to identify the value IMGs put on these supports, and any additional recommendations they had for institutions and IMG peers for harnessing change and enhancing the IMG experience.

## Methods

### Study design and setting

The aim of this study was to explore what supports IMGs are currently receiving in Australia, their perceived utility and IMG-led solutions for improvement. The study formed part of a larger PhD body of work, exploring the journeys and lived experiences of IMGs in Australia. Four other papers from the wider study have been published, examining IMG experiences of inequity [17], common lived experiences and perceptions [18], research participation [19] and mental health [20].

This study employed a sequential explanatory mixed-methods (SEMM) design, and therefore had two parts- 1) cross-sectional online survey and 2) semi-structured interviews. The SEMM design was intentionally chosen as it enabled broad exploration of ideas firstly through the survey, and then seek deeper understanding of underlying perceptions, by interviewing a sub-sample of participants [21]. SEMM design was also chosen as it served to strengthen data validation, by presenting evidence through different data sources which could be used to triangulate the findings and explain findings more thoroughly [21]. The online survey was distributed to participants across Australia between 13 October 2023 to 31 December 2023 (11-week period), with reminder emails at the 5 and 9-week marks. The survey was created using REDCap (Research Electronic Data Capture) tool hosted by Hunter Medical Research Institute [22,23]. Individual interviews were conducted by the primary researcher by either telephone, teleconference or face-to-face, between 6 February 2024 to 18 April 2024. ‘Low risk research’ approval was granted by the College Human Ethics Advisory Panel University of Newcastle: H2022-0392; with access request granted through Hunter New England Health Human Research Ethics Committee (HREC): AR20230405_Nair and Central Coast Health HREC: 0323-024C. Consent was obtained by all participants as approved by Ethics; implied consent for survey participants and written consent for interview participants.

### Participants, recruitment and sample size

A non-random sample of IMGs living in Australia, irrespective of employment status, were invited to participate in the survey and/or interview. Any person living in Australia with past or current intention to practice in Australia and holding a primary medical qualification external to Australia was eligible to participate. Participants were recruited through a variety of avenues (social media groups, courses and workplaces frequented by IMGs, a national college and snowballing) to maximise opportunity for the survey-link reaching a wide range of participants. Recruiting avenues are deliberately unnamed in this paper to protect identities of both the agencies and IMGs. As there is no openly available information for the number of IMGs living in Australia, we aimed for a conservative sample size of 200 participants, based on the number we expected to recruit and expected to provide sufficient variation in demographics and outcomes, enabling the collection of meaningful initial descriptive data from the population. Survey respondents interested in participating in the qualitative phase of the research were forwarded further information and were invited to interview. Following written consent being obtained, interview appointments were arranged and conducted at mutually agreed times and locations/modalities. Snowballing methods drew five additional interview participants to the study. Interview sample size was determined by the number of written consent forms returned to the research team within the specified 3-month time period, aiming for a minimum of 20 participants, unless data saturation was achieved earlier.

### Instrument design and data collection

a) Survey data collection: Twenty-six exploratory items relating to support were posed to participants, within three sections (see S1 Appendix). Support items were chosen based upon proposed solutions to IMG inequity which were summarised in a preceding scoping review [12] together with input from a small team of educational researchers with expert knowledge in content and methodology. The online survey was tested for usability and suitability with ten IMGs prior to dissemination, ensuring face and content validity. The questionnaire underwent several iterations prior to the final version being distributed to study participants. Items included: 1) two questions asking participants to rate on a 5- point Likert scale (“strongly agree” to “strongly disagree”) their satisfaction with current supports in the workplace and by institutions; 2) 4- point Likert scale rating of utility (“not useful at all” to “very useful”) for twelve suggested supports for IMGs; 3) a table presenting twelve types of supports, asking participants to checkbox which supports they had used or received since being in Australia. In addition, four open text questions allowed survey participants to freely comment and make suggestions about processes such as the AMC (Australian Medical Council) exam, registration and working conditions in Australia. The estimated completion time was 20-minutes. Other than the first two survey questions which established eligibility, each survey question thereafter was non-obligatory for completion. This was to encourage participants to prioritise sections if they wished to. Survey data was collected and managed using the REDCap tool [22,23].
b) Interview data collection: The six-question semi-structured interview guide was modified following analysis of the survey, to maximise exploration and explanation of the survey results. To explore supports, we asked IMGs to “Please share any ideas you have for bettering IMG experiences in Australia” which included probing for institutional, workplace and interpersonal support and personal advice. With consent, audio data recorders were used to capture interview data, together with field notes.

### Analysis and reporting

The three study sections were analysed separately prior to triangulation: a) quantitative survey data, b) qualitative survey data (open responses) and c) qualitative interview data. Thematic analysis was undertaken on the qualitative survey and interview data, as guided by Braun and Clarke’s principles [24].

a) Analysis of quantitative survey data was conducted using Stata version 17 [25]. Descriptive analysis was chosen to understand the survey data, as the study was exploratory in nature.
b) Analysis of qualitative survey data (open responses) and interview data: NVivo [26], a qualitative data software tool, was used to sort the data and facilitate coding. A coding tree was refined by two researchers with qualitative experience. One of the researchers independently coded common ideas before developing overarching categories and identifying preliminary themes in the data. Themes were discussed and reviewed with a second researcher prior to finalisation. Full analysis of the quantitative and qualitative survey data was undertaken before undertaking the interview phase of the study. Findings of the survey informed questioning in the qualitative interviews, as per SEMM approach.
c) Analysis of qualitative interview data: Interview data was transcribed by the primary researcher onto Word documents, within 48 hours of recording. Data cross-checking was performed on a random sample of 4/36 (~10%) recordings/transcripts, by a second researcher to ensure quality and research integrity was maintained. Following this, two researchers with experience in qualitative research created a common codebook and co-coded 10% (four) transcripts to ensure uniformity of coding before the remainder were singularly coded.. Thematic analysis was undertaken primarily by the primary researcher, with review and oversight by two researchers with expertise in methodological research. Common themes from the interview data were identified, reviewed, compared and triangulated with the survey data (quantitative and open responses) in order to make sense of the data in a meaningful way for reporting below.

This study adhered to the Good Reporting of a Mixed-Methods Study (GRAMMS) [27] standard and the checklist is included in S2 Checklist. We report the combined main results in this paper to describe 1) Supports experienced by IMGs in Australia- 1a) Formalised institutional or workplace support, 1b) Informal networks and community support; 2) How institutions can better support IMGs- 2a) Improve operation of bureaucratic systems, 2b) Institutions can better mobilise IMGs by supporting integration and skill development, 2c) IMGs call for changes to reduce discrimination and exploitation risks; 3) How IMGs can better support themselves- 3a) Attitude and adaptation are important, 3b) Be prepared and seek advice early, 3c) Consider life stages.

### Stance and reflexivity

We approached this study with a stance of pragmatism, exploring the topic and constructing ideas from subjective and objective information. Two authors (SJRH and BRN) are IMGs and work closely with IMGs in clinical and educational employment. The primary researcher (SJRH) has worked in Australia for twenty years, across four states/territories and in a variety of clinical and non-clinical roles. To maintain objectivity in the conduct of the study, SJRH regularly met with non-medical co-researchers (KF and BSMA) to discuss and debrief regarding results and interpretation. Procedures such as member checking, notetaking and exclusion of interview participants closely known to the primary researcher, were undertaken to ensure research was undertaken and analysed with integrity.

## Results

Of the total 286 participants in the survey sample, up to 192 participants completed the ‘supports’ section, indicating ~67% response rate. Participant numbers per section vary due to the non-compulsory nature of the survey questions. A broad range of IMGs across Australia participated in the survey, working in 17 different specialties, and obtaining their primary medical qualifications (PMQs) from a total of 46 countries, with various training and employment backgrounds. Participant characteristics are summarised S3 Table. A minimum of three participants from each state/territory participated in the survey. 134/191 (70.2%) participants indicated that they had received or used at least one of the supports listed in the survey since being in Australia. Open-ended responses predominantly expressed themes around dissatisfaction with current bureaucratic processes and solutions to common challenges.

The interview participants were predominantly drawn from the survey, with an additional five participants from Trinidad and Tobago, South Africa, Bangladesh and Brazil. Reported demographics of interview participants largely reflected those of survey respondents. Interviews were conducted by telephone (21/36), teleconference (14/36) and face-to-face (1/36). The majority of interviews took 30–60 minutes, although three extended to almost 90 minutes.

### Supports experienced by IMGs in Australia

IMGs reported receiving a variety of supports both inside and outside the workplace, although the degree and source of support was highly variable.

#### Formalised institutional or workplace support.

“WBA program was worth it, as I got honest face-to-face feedback and was actually interviewing real patients with real issues, which meant that signs could be identified” [survey participant open response].

Almost half of the survey participants were satisfied with the current support they received from their workplaces (98/197; 49.8%); a lesser number were dissatisfied (31/197; 15.7%); and a sizeable proportion were neutral (68/197; 34.5%). One hundred and thirty-four participants reported using at least one workplace support since being in Australia. The most reported workplace support received/utilized were cultural competency induction courses (46/134, 34.3%), followed by a socially inclusive environment (40/134, 29.9%) and established mentoring/ peer support (31/134, 23.1%)(see data in S4 Table).

Several interviewed IMGs reported highly valuing any support which had been offered to them by institutions. Some IMGs reported general support from hospital administration coordinators, particularly in certain rural hospitals, where IMG recruitment was commonplace. Such support was reported to be helpful for orientation, integration and settling. Some IMGs reported practical help and support from individuals within certain departments. Some IMGs reported good support from supervisors and consultants, particularly with direction around treatment guidelines. One IMG said: “I worked independently, but I had someone who was constantly at hand who could help me, plus they were also checking that I was doing the right thing” [Interviewee #16; PMQ: South Africa]. Some seniors were helpful with non-work-related matters, such as references for rentals and assisting with transport. Although not often reported, those who had mentors told how helpful they were regarding debriefing, feedback and career advice. One IMG who was being bullied reported excellent support from her manager, who swiftly actioned the complaint: “… the manger I complained to was readily available, and she was interested immediately…That was the best support…like, I told her today, and tomorrow she did it. Just like she used a magic wand or something” [Interviewee #9; PMQ: India]. Two IMGs explained how they appreciated constructive and polite feedback about the clarity of their accent and improvement of their language skills. One IMG explained: “He told me that ⅹⅹⅹ you are speaking too fast, and you have an accent. You should try to [slow down]…, makes sense… it is not my native- English. But I am trying. I try to speak slowly. And I think it [feedback] is a good one” [Interviewee #17; PMQ: Egypt].

General (non-IMG specific) education sessions and workshops for junior doctors were reported as useful by several IMGs[e.g., Aboriginal cultural awareness, technical skills and resuscitation etc]. A few IMGs reported that *IMG-specific* structured faculty-based programs were helpful for integrating IMGs. However, these only occurred at a few workplaces. Workplace based assessment (WBA) educational courses were considered useful by several IMGs for orientation to the Australian healthcare system and also for helping IMGs understand clinical expectations in their roles. One IMG explained: “… [The WBA] orientation days were really good. They would explain how Medicare would work. And all these little elements of Australian healthcare- and it’s specifically focused on IMGs [Interviewee #27; PMQ: South Africa]. Several IMGs reported that 6-week Observerships were valuable for orientating IMGs to the hospital system, understanding expectations and roles, and also familiarisation with English language. One IMG explained: “….when we go and observe, we get to see everything. We get to see the communication, how the doctors speak to the patients, how the patients are, how the system works, runs- everything!” [Interviewee #18; PMQ: India]. IMGs gave mixed reviews about bridging courses.

Other institutions reported by IMGs to be useful were – a)recruitment agencies and locum agencies, who helped arrange visas and jobs and b)the Australian Medical Association (AMA), who helped advocate for IMGs experiencing bullying in the workplace.

#### Informal networks and community support.

“My workplace seems to be very inclusive and supportive across different cultures. I feel support to IMGs in this regard is vital to ensure good mental health and wellbeing” [survey participant open response].

Informal networks and community support were explored in the interviews and open-ended survey responses. Interviewed IMGs reported receiving various informal supports within the workplace, or out in the community, which helped transition to life in Australia as a new IMG migrant. Many IMGs reported gaining strong support from other IMG peers, either local to their area, or interstate. Having a contact person or social group, particularly from their own country was helpful for IMGs, giving reassurance and guidance. IMGs congregated from work, or examination preparation study groups and reported bonding over similar life stages and experiences. One British IMG said: “[My main social support] would be the UK group…Great Britain. Because where they are in their career, is the same as well. They may be all wanting to get out of the NHS, for a while, get some experience, tee up an application for when they get back. So, they’re actually all doing the same thing. They’re all…true peers, in that sense” [Interviewee #12; PMQ: UK]. Social media groups were reported as useful for unofficially seeking advice and encouragement from IMGs with various backgrounds and experience. Several IMGs reported that a supportive workplace and general kindness from ward staff helped them feel included and valued, thereby helping integration. One interviewee said: “The workplace culture where I’m working right now is really great. It helps IMG to settle, physically…mentally, and to understand the system” [Interviewee #3, PMQ: Sudan]. Some IMGs received financial and/or emotional support from family or friends who had already settled in Australia. Those who came with spouses generally reported great support both financially and emotionally, particularly if spouses were of medical background themselves. Practical help and support was received from community members, such as neighbours, religious groups, cultural groups, and school parents. One IMG explained: “All the systems really blocked us, but the people helped us enormously.... There were all sorts of people [in the community] who chipped in... Connections with people that we worked with...[for example] the spare car of the principal where we enrolled our kids…” [Interviewee #2; PMQ: UK]

Lastly, several IMGs reported the benefits of a positive mindset- self-talk in reassuring themselves of their own abilities and encouraging self-confidence. Some IMGs reported their attentiveness to self-care such as healthy eating, exercise, spiritual health and personal resilience as important to support and strengthen themselves physically and emotionally. One IMG explained: “I try to eat healthy; I go to the gym; I go for walks. I try to keep myself. I pray to God. I talk to my parents every day. I talk to my friends a lot in India. I call them, like my therapy. So, I try to be as happy as I can for my children’s sake” [Interviewee #33; PMQ: India].

### How institutions can better support IMGs

IMGs identified and proposed several ways of better supporting new and current IMGs in Australia.

Survey participants were asked to rate suggested supports on a four-point Likert scale ranging from not useful to very useful (see data in S5 Table). According to the survey participants, the top ‘very useful’ proposed supports for the future were:

1) Streamlining bureaucratic processes (126/189 (66.7%))
2) Modification of assessment requirements based on recognition of previous qualifications/experience (125/192 (65.1%))
3) Consultation of IMGs in qualification recognition/training/assessment (117/188 (62.2%))
4) Recognition and matching of previous qualifications/ experience to future allocated jobs (118/190 (62.1%)) and
5) Establishing departments providing ongoing support/advocacy for IMGs (112/188 (59.6%)).

Other highly rated proposed supports were: Established mentoring and peer support systems (111/187 (59.4%)) and fostering a socially inclusive environment in the workplace (102/189 (54%)).

#### Improve operation of bureaucratic systems.

“I wish there was an organised body that we could get practical advice from before we start the process, so we don’t get surprised or overwhelmed down the road with all the obstacles, requirements, fees etc” [survey participant open response].

Details exploring and identifying improvements to bureaucratic operations are reported from the survey qualitative data (open responses) and interview data only. Improvements to the functionality and efficiency of current bureaucratic operations were highly recommended by IMGs, as expressed by open-ended survey responders and interviewees. Several IMGs reported that the currently tedious and expensive processes of examination, registration and training scheme access were “frustrating and turning me off specialising in Australia” [survey open response]. One interviewee said: “Like the paperwork, coming here, and the AHPRA now…I have said to my [partner] multiple times already like...shall we just go home? Like this is just such a burden” [Interviewee #29; PMQ: Netherlands]. Institutions which IMGs reported could benefit from change were the Australian Health Practitioner Regulation Agency (AHPRA), the Australian Medical Council (AMC), employers, specialty colleges and government agencies (e.g., Home Affairs and Services Australia). Recommendations included the centralisation/unification of services to enable better communication and sharing of information between institutions and streamlining and coordination of processes. Interviewed IMGs proposed that such changes would reduce duplications, thereby saving time and financial costs to both IMGs and institutions. IMGs reported that a clear, concise, current and complete ‘roadmap’ information guide should be provided via a centralised website to address each IMG journey stage. This should preferably be explained using a video medium to aid understanding with links to relevant resources and contacts. One interviewee explained: “There can [should] be a website for IMGs that showing from A to Z. Because there can be people who don’t know anything, and how to start… a site for studying section, and taking exam section, and can be a seeking employment section. From the studying section, can introduce the textbooks, and attending courses, which course are available, some information. And to exam...how to prepare exam maybe for someone else, and how to register for example. They can [currently] find information on AMC website as well, but information [is] all scattered. If you go to AMC website, it very long. It’s difficult to find what you want and how to read everything. Just one piece of information. So, can [should] be concise as well” [Interviewee #25; PMQ: China]. IMGs also proposed that reduction in costs, or provision of budget friendly payment plan options would hasten IMGs joining the workforce earlier. Importantly, IMGs reported that institutions need to be more transparent about financial costs and realistic prospects (e.g., career options, exam pass rates and requirements, immigration rules etc), in order for IMGs to make truly informed decisions and be better prepared upon arrival.

#### Institutions can better mobilise IMGs by supporting integration and skill development.

“I think having less stress on securing registration and providing less assessments will make me more focused on hospital work and skill development. During working hours, it is difficult to manage studies and work life balance” [survey participant open response].

Details exploring and identifying improvements to IMG mobilisation are reported from the survey qualitative data (open responses) and interview data only. IMGs suggested various supportive ways for institutions to empower and integrate IMGs, thereby mobilising a fit-for-purpose workforce. Many IMGs recommended disbanding the current AMC assessment process and re-directing costs into mainstreaming direct clinical assessment with clear marking schemes and feedback. One IMG said: “[The] mechanism of assessment [standard AMC exams] is so impersonal. And I think the report of my colleagues is far more important…the fact that consultants have seen me working and have trusted me to look after patients with these complicated conditions…that’s far more important than me passing the MCQ” [Interviewee #36;; PMQ: South Africa].

The Workplace Based Assessment (WBA) or similar, was suggested as a superior assessment model which provided synergistic benefits- supporting workforce entry, providing direct feedback and equipping IMGs with workplace skills in the local healthcare system. One interviewee explained: “… people who do WBA, they *work*. They work! … they’re knowing the system, they’re studying, they’re doing their assessment inside the system...That’s perfect. When they finish, they have general registration, and they can continue. And the work that they did… is going to count as experience when they [are] applying for a job a year later” [interviewee #19; PMQ: Italy]. Another IMG recommended the promotion of WBA program over the standard AMC examination: “Increase the capacity for WBA in various hospitals to both motivate IMGs to less popular tertiary hospitals and enable more people to enter the pathway via a less stressful process” [survey participant open response].

Several IMGs suggested that institutions should facilitate and accredit workplace transition programs such as observerships, bridging courses and clerkships. Several IMGs also recommended mobilising the workforce by making changes to the current requirements for general registration. Several IMGs recommended that completion of AMC assessments should automatically permit general registration, irrespective of job offer. One IMG wrote: “The IMGs should be given opportunities to work at first... IMGs are doing nonmedical jobs for livelihood despite AMC1 [MCQ exam]. There should be provision of getting registration after the exam, rather than after getting a job offer” [survey participant open response].

Many IMGs recommended assessment and/or recognition of their prior experience, with suitable advice provided for job options to suit their skill set. Furthermore, IMGs recommended that a review of past experience should be conducted on a case-by-case basis, and that the employment awards should be standardised Australia-wide. One IMG wrote: “recognition of prior work needs to be legislated or there needs to be some standard way that previous experience is assessed. As a PGY6, I am currently paid as a PGY2. How is that legal?” [survey participant open response]. One interviewee further explained: “I’m asking just for a mechanism by which my experience can be assessed. And if after process like that, they still feel that no, HMO2 or HMO3 is appropriate, I would accept that. But I just want the opportunity to have someone actually look at that… I’m just asking that the mechanism should exist” [Interviewee #36; PMQ: South Africa].

Many IMGs recommended a clear, dedicated advocacy body enabling IMGs to seek widespread assistance, in and outside the workplace. Additionally, several IMGs recommended a dedicated go-to liaison officer/ coordinator at health district level, who can provide immediate support and education about IMG rights, help navigate work and non-work-related issues, and provide individualised career guidance based on experience. Some IMGs recommended that this role be taken on by a separate IMG-specific department within the organisation. One interviewee said: “I think the hospital needs to have a like a…well trained staff…like a group of staff members who are familiar with [IMG] problems and can help you navigate them” [Interviewee #36;; PMQ: South Africa].

Many IMGs recommended formal mentoring, peer groups and buddy systems as avenues to connect IMGs with others who can give sage advice and encouragement on various stages of the IMG journey. One IMG wrote: “an Australia-wide peer-[to]-peer mentoring system would be useful to empower IMGs to raise complaints and navigate the process of adapting to a very different system” [survey participant open response]. Some IMGs reported that having an IMG mentor with lived experience would be most suitable. On the other hand, a few IMGs reported that having an Australian mentor might help Australians appreciate IMG culture and background, promote unity and help integration.

Many interviewed IMGs recommended that institutions provide ongoing teaching sessions, courses or workshops for IMGs which explicitly focus on Australian culture, acceptable and unacceptable workplace behaviours (e.g., bullying), clinical responsibilities and expectations, health system differences (e.g., Pharmaceutical Benefit Scheme, Medicare, National Disability Insurance Scheme etc), patient-centred communication, IMG rights, workplace norms and roles of staff, patient ethics, LGBTQIA+ health, allied health and language courses (including phraseology, slang and colloquialisms). One IMG recommended that education sessions be conducted as ‘IMG-only’ sessions, run by IMGs, “only for IMGs…where you wouldn’t feel so intimidated to say things, make mistakes and all that” [Interviewee #10; PMQ: India]. Several IMGs explained that explicit instruction about workplace culture would be helpful, to prevent IMGs invertedly offending others or presenting themselves unfavourably. One IMG suggested directed orientation: “…we need to be told that it’s a different health system here, where you speak up and you communicate rather than being silent… like what work culture looks like in Australia” [Interviewee #8; PMQ: India]. Furthermore, one IMG recommended reminding established IMG consultants of the flatter hierarchy in the Australian medical workplace, including acceptable behaviour when interacting with junior staff. Accredited bridging courses with an Australian focus were also recommended.

IMGs recommended that specialist colleges take a more active role in supporting IMGs, particularly those placed in rural locations, to reduce inequities between training doctors in education and career outcomes, e.g., funding exam teaching courses. One interviewee said: “…we need to make a clause that…we should do a couple of terms in a tertiary hospital, to get exposure. That is very essential… for exam purposes…and… [to] boost your confidence.” [Interviewee #10; PMQ: India]. Another IMG summarised the need to support rurally placed doctors: “And there’s a reason why the Australians won’t take the jobs. And we need to actually make the jobs better. And stop relying on just importing people who have very few rights. But if we’re going to import them, then we need to actually be decent to them, and try and support them” [Interviewee #2; PMQ: UK].

Lastly, several IMGs suggested that early constructive feedback from co-staff be given in a timely and polite manner. IMGs explained that this would help IMGs to quickly action areas of improvement and avoid further unintentional errors, particularly around communication. One IMG said: “….just tell me what to do and I will do it!… Just please let me know! … Yes, if you just tell me, I will do it”[Interviewee #20; Egypt].

#### IMGs call for changes to reduce discrimination and exploitation risks.

“It requires a total Australia wide cultural overhaul. There’s a reason the new consultants advertised themselves as ‘Australian graduate’. It’s an advantage**”** [survey participant open response].

Details exploring and identifying discrimination reduction are reported from the survey qualitative data (open responses) and interview data only. Several IMG interviewees and open-ended respondents reported that some current bureaucratic systems facilitate discrimination and that systemic changes are necessary in order to reduce risk of exploitation and encourage fair conduct. Several IMGs recommended revision of the current immigration visa restrictions, and removal of the link between visa and employer, to reduce risks of employer exploitation. One IMG said: “I think the very first step is for them not to hinge the visa on any private person. … if the government is going to sponsor an IMG, give them a visa, to come and work in this country as a doctor; then the government should be in charge of the visa… The visa and the AHPRA registration are very strong tools to exploit” [Interviewee #7; PMQ: Nigeria].

Review of the moratorium restrictions (geographical and financial) and permanent residency requirements were also recommended. Some IMGs suggested that IMG representation and inclusion in policies and policy making committees would help keep institutions accountable and fair. Several IMGs reported the need for fairer work conditions, in terms of rostering of shifts, rotations and distribution of work, and equity of pay for level of experience and jobs performed. Many IMGs reported that disparities in education, conditions of work and pastoral care for those working in non-metropolitan locations should be considered and addressed, in order to maximise success in exams, training and career progress. The overrepresentation of IMGs in these locations was reported as a particular priority for colleges and supervisors to address.

Several IMGs recommended prioritising workplace harmony. IMGs recommended non-IMG staff undertake mandatory cultural training sessions to educate about respecting cultural differences, diversity, implicit biases and addressing racism. One interviewee pointed out: “There is racism-…to fix the problem, you need to admit and acknowledge it. There is a problem, and the next step, you have to work on it” [Interviewee #20; PMQ: Egypt].

Some IMGs suggested promoting a culture of safety and respect by promoting a strengths-based approach rather than focussing on deficits, e.g., focussing on the skills and expertise that IMGs bring. Furthermore, a few IMGs recommended opportunities for IMGs to safely share their life stories. One interviewee said: “I think that just sharing the humanity and the background of these people who come and seek a new future here, a new life…I think would be tremendously humbling and educational to everybody to listen to [Interviewee #2; PMQ: UK]. Another interviewee explained: “… it would be wonderful if in the orientation, [IMGs] are given an opportunity to tell their story. Because I have come across the most astonishing stories, that if anybody heard them, it doesn’t matter how inept they are with the current system, people would just see them in a different light, and it would humanise why this person is here” [Interviewee #21; PMQ: UK].

### How IMGs can better support themselves

“I still think IMGs who’s coming here, is one of the brilliant in their country. So, you are trying to be brilliant. We are like a diamond, but you always need to polish yourself…yes, polish yourself, and always you will keep going” [Interviewee #17; PMQ: Egypt].

Reports of how IMGs can better support themselves were explored in the qualitative interview study only.

#### Attitude and adaptation are important.

Many interviewed IMGs gave suggestions encouraging their IMG peers to harness their own agency. IMGs recommended that taking initiative, having an enquiring attitude and resilience were important assets for overcoming adversity and forging successful careers. Perseverance was especially noted to be important in the early adjustment phase of migration. Several IMGs suggested “embracing change” and adjusting or adapting to their environment for success, for example, modifying their accent to improve understandability or using terminology commonplace in Australia. One interviewee said: “… be open, be honest. Learn about the new culture, but also if you are unsure about things, just ask. I think a lot of IMGs feel a bit scared and they want to please. They don’t want people to know their vulnerabilities or their lack of knowledge and then that can be a hindrance in the long run. So just ask and embrace the change” [Interviewee #24; PMQ: UK]. Another IMG advised: “if you’re willing to stick your neck out,…then you’ll always be pleasantly surprised with all the opportunities that come” [Interviewee #11; PMQ: India].

One IMG recommended regular self-reflection as a useful assessment tool. Self-appreciation for skill set was identified as a way to improve self-confidence and combat ‘imposter syndrome’. Several IMGs recommended that informal chatting with ward staff could help IMG peers integrate better, with the secondary gain of English language improvement. Finding common interests with people in and outside the workplace was recommended as an avenue to help socialisation. One IMG suggested: “…branch out.. Make friends with Australians and locals. That certainly has helped me” [Interviewee #31; PMQ: Germany]. Several IMGs identified that the “quiet achiever” disposition and lack of portrayed self-confidence was detrimental to IMG success in Australia. Hence many IMGs advised that their IMG peers should be more outgoing, show confidence and ask for advice and help when uncertain, without fear of judgement or repercussion. One IMG Consultant said: “If you can’t do something, let me know, …no big deal- we just figure it out” [Interviewee #12; PMQ UK]. They went on to explain that for IMGs from some cultures, there may be a “] price of admitting that you might not know something in front of other people …embarrassment, or humiliation, or being shouted at,...[but] if you can’t make that step, you’ll always be behind” [Interviewee #12; PMQ: UK]. Two IMGs specifically mentioned maintaining strength and resilience in the face of racism. One IMG shared this advice: “…do you want to succeed, or do you not want to succeed in this system? So go above and beyond. Be better than everybody else. And look better, look smarter, look them in the eye…train yourself up in all these things. And you do have the extra hurdle…and it’s not a level playing field. But…you’re in this far- what are you going to do? …fail? And tell yourself the whole system is racist and… fail? [Interviewee #21; PMQ: UK].

#### Be prepared and seek advice early.

Interviewees also suggested IMG peers proactively learn details of their local health system (including guidelines), seek training in deficits and seek feedback. One IMG said: “stop feeling scared…believe in yourself… try to know the system, and going through the policies and guidelines really help you know how to approach a clinical situation” [Interviewee #10; PMQ: India]. Another IMG recommended: “…don’t look at it like a pressure. Look at it like you upskill yourself and let them know that even if I went [came] from different… [PMQ country]…. I still learn medicine in a good way, and I will do my best” [Interviewee #19; PMQ: Egypt]. IMGs reported that this would help improve self-awareness and empowerment. Several IMGs recommended being aware of personal rights in the workplace, as a means of reducing risk of exploitation and bullying. One interviewee said: “I could stand up for myself, because I had information” [Interviewee #5; PMQ: Sweden]. Another interviewee implored: “please tell people that they have help available. Please feel free to take it. Please don’t worry about the consequences, don’t be bullied…[Administrators] always tell ‘help is available, help is available’, but they should be telling in full sentences that ‘help is available, and we can assure you that you don’t have to worry about any bad repercussions’” [Interviewee #9; PMQ: India]. Advice to “stand up for yourselves” was frequently cited, as were suggestions to actively seek out mentors and other supportive people.

#### Consider life stages.

There was mixed advice about when best to migrate- some IMGs recommended coming to Australia soon after graduation, as not to “waste so much of their years” [Interviewee #18, PMQ India], whilst others recommended migrating with more experience, anticipating better access to jobs and programs such as WBA. A survey open ended respondent highlighted the need for IMGs to avoid a 3-year clinical practice gap to avoid difficulties with registration requirements: “I would like to advise other IMGs to have minimum as possible gap years. I found it very hard when you are out of practice for more than three years.” [survey participant open response].

Some IMGs recommended avoiding having a family before completing fellowship exams, to reduce distractions from competing responsibilities: “If you have responsibilities, you can’t focus on your studies. If there was one advice- get your exams, get everything under your belt, and then you decide to settle in life. Because if you get settled too early in life, you have all those responsibilities, and your focus in life will not be there” [Interviewee #11, PMQ: India].

Another IMG reported the presence of family as a way of maintaining perspective.

### Triangulation of findings

We triangulated findings by combining the methodologies and analyses of the quantitative and qualitative components of our study. The quantitative component provided an overall appreciation of what workplace supports were experienced by IMGs in Australia, whilst the data from the qualitative study components delved into how these workplace supports were important, and also into broader informal networks and communities support. Following analysis of the survey data, we identified several areas for further exploration in our follow-up interview study. The interview question regarding supports were guided to explore the reasons behind IMG-led recommendations and also discover peer advice.

Generally, there was congruence between the qualitative and quantitative data. For example, the high response for recommending streamlining bureaucratic processes (66.7%) in the survey results was confirmed by qualitative results which explained how changes to current systems could be made to mitigate IMG challenges. Also, quantitative results identified a high need for recognition of previous qualifications and experience in the modification of assessment requirements (65.1%) and future allocated jobs (62.1%), which was strongly supported by the qualitative data. The relatively low proportion of survey participants (23.1%) receiving mentoring support was reflected in the qualitative data. However, the desire and recommendation for such support was prominently shared in the qualitative data, indicating the value on a personal level. This was most strongly described in the interview data, although less pronounced in the quantitative figures (59.4%) which prioritised changes needed to bureaucratic operations and assessment.

## Discussion

This study identifies and summarises an array of solutions to improve support for IMGs in Australia, from an IMG lens. Our study is useful as it describes reflections of IMGs on the supports which they have had to date, and their utility. Furthermore, our chosen methodology has provided a depth of understanding into the value of certain supports and changes that can be made- insights which are not seen elsewhere in the literature. Our results are novel in that they provide not only direction for institutions and workplaces, but also key insights into the reasons behind why such changes are necessary. Additionally, our results offer leverage and advice for IMGs as individuals, by sharing wise learnings from IMG participants themselves. Data in S6 Table summarises IMG-led recommendations for institutions and IMG peers.

Our findings of bureaucratic criticisms are not new to the literature. Worldwide, bureaucratic policies have been reported to inadequately support IMGs entering the workforce [3,28,29]. Institutional hindrances have been reported to impact IMG personal journeys as well as obstructing service delivery for the wider population [3,30,31]. Kruk’s recent Independent Review of Overseas Health Practitioner Regulatory Settings in Australia paralleled our findings, proposing many similar recommendations such as streamlining and centralising bureaucratic services, improved transparency, better recognition of prior experience and revisions to institutional assessments and processes [3]. Outside of bureaucracy, IMGs may encounter several other challenges related to acculturation and adverse work conditions which may impair career attainment and health [12,32]. Migration stresses may particularly affect those emigrating from countries with socio-political instability. Therefore, the attention of dedicated staff and services who are equipped in assisting IMGs is needed. Several IMGs in our study pointed to current immigration and work policies in Australia which place IMGs at risk of exploitation, negative and unfair treatment Detailed reports of structural discrimination and other forms of inequitable treatment impacting IMGs is described in our wider research [17]. Both the American Medical Association and the Australian Medical Association recognise additional hurdles impacting IMGs, such as visa and licensing restrictions, and strongly advocate for fairness [31,33,34]. Urgent review and attention by institutions to such policies is warranted.

Since our study was conducted, Nwankwo et al published a scoping review mapping strategies of IMG support, examining 30 studies from UK, Australia, Canada and USA between 2010–2023 [35]. The nature of support strategies identified were broadly grouped into educational strategies (e.g., orientation, training workshops, clinical attachments/observerships) and social strategies (e.g., social networking, peer support, buddying, support from family/friends, pastoral and administrative assistance-both inside and outside the workplace, e.g., accommodation, schooling for children etc). Furthermore, Nwankwo highlights the need for robust evaluation of the intervention/support outcomes (e.g., transfer and retention of knowledge for educational strategies) [35]. Our findings support those identified by Nwankwo, plus agree with the recommendation to create an environment where IMGs with specialist experience share their expert skills and knowledge gained internationally, thereby enriching the workplace.

Experience sharing was an important aspect of support highlighted by our participants. Peer support groups are an informal and flexible intervention to provide safety for doctors discussing work and personal difficulties [36]. Mentoring is a well described intervention for individuals in managing their own intellectual capital and performance [37] and is offered to members of several specialist colleges in Australia [38–40]. However, IMGs who are not affiliated with such institutions may be missed, as seen in our study. Mentor training to effectively support IMG needs may be required [41]. Therefore, a more unified approach to capture all IMGs (employed or non-employed) seeking mentor guidance at different stages in their career and migration journey, would be beneficial.

Indeed, our study participants described the need for IMG-specific support and advocacy groups to be established in Australia. Although institutions in Australia have programs, groups or case officers to support IMGs [42–44], currently, there is no unified national body where *all* IMGs can readily access independent advice on a range of IMG-specific issues in one location. We propose that such a body would assist in bridging the gap between institutions and workplaces, whilst also providing direct support to IMGs, particularly those who are unemployed, or struggling to achieve meaningful employment as doctors in Australia.

Although the value of constructive feedback has been widely accepted in the medical education arena, it is somewhat surprising that several of our study participants reported sub-optimal experiences with receiving feedback in a timely manner. This may be due to perceived or real cross-cultural communication complexities and hesitations, between both deliverers and receivers of feedback [45]. Strategies to help deliverers of feedback to confidently communicate feedback in a culturally safe and respectful manner may be useful for staff working with IMGs. Similarly, other aspects of cultural safety should be considered to enhance IMG-welcomeness in workplaces. The recommendations given by our participants to include IMGs through a strengths-based approach in the workplace is novel. We found no other articles in the literature which give clear direction about how workplace harmony can be afforded to IMGs. Indeed, this is an area which requires attention and further study.

Our study supports others who have found the value of a solid orientation program for IMGs educating about the host country’s health system [46,47]. Orientation should be dynamic, comprehensive, ongoing and considering workplace and non-workplace needs [36]. Saxena’s Australian qualitative study of 19 IMG participants found IMG-identified learning needs in communication skills, legal and ethics situations, medical knowledge specific to Australian context, medical roles and role-play examination techniques [47]. Our results strongly support Saxena’s findings, adding the importance of educating IMGs about workplace culture (including different responsibilities and expectations of staff and hierarchies), patient centred care, role of allied health, and LGBTQIA+ health.

The advice given by participants in this study for other IMGs is empowering. There is little in the literature which explores IMGs as active participants in enhancing their own transition process of working in a new country. Kehoe’s realist synthesis of IMG interventions, which was heavily based on literature search of discussion papers and program evaluations, reported self-engagement of IMGs as an important factor in self-aiding the transition process [8]. Huijskens’ qualitative study of 32 IMGs in the Netherlands supported that personal qualities of persistence and flexibility were important for career progression [48]. Capacity to change, motivation to engage in transformative learning and self-awareness were identified as valuable factors for successful transition [8].

### Implications for practice

Our study increases awareness of IMG-recommended supports, and provides specific, practical and actionable suggestions. Institutions and non-IMG colleagues may consider incorporating this consumer-voice evidence into reviews and policies impacting IMGs. Also, direct advice provided from IMGs in this study serves to instruct and better prepare IMG-peers whilst also providing a sense of solidarity and encouragement.

Furthermore, our research identifies that the responsibility to support IMGs falls with a variety of stakeholders. S7 Fig summarises the recommendations derived from this study- the different responsibilities of institutions, workplaces and IMGs, in addition to postulation of a newly formed IMG advocacy body. Stakeholders would do well to adopt a cooperative and multipronged approach to supporting IMGs.

### Strengths and limitations of study

To the best of our knowledge, this is the first study that investigates IMG supports from an IMG perspective, with a mixed methods approach to deeply explore the data. The mixed methods approach allowed ideas conceived in the quantitative study to be delved into more comprehensively in the qualitative interviews. The insights gained are both novel and rich and have the propensity to inform further attention and study by researchers and policymakers in the IMG arena. The solutions we have identified may be a helpful base for host countries who seek to attract international skilled health professionals.

However, the study is not without its limitations. The study used a sample of IMGs located in one country and therefore results may not be representative of all IMGs globally. Potential study bias exists in the form of participation bias and social desirability. The dearth of other similar studies in the literature limits generalizability of our results. The study does, however, serve as a platform for larger, more robust studies in the field. Indeed, international studies are recommended to gain a fuller picture of how IMGs can enhance their experiences across borders. It is worth noting that the IMG interest demonstrated in participation in this study is an indication that a portion of IMGs in Australia wish for future change.

## Conclusion

Enhancing the IMG experience is important for safeguarding the IMG workforce in Australia. IMGs can be better supported by institutions and policies which promote integration, cultural safety and fair work conditions. IMGs can also enhance their experiences by harnessing their own agency. Further exploration and attention is needed into improving conditions for this understudied yet essential workforce.

## Supporting information

S1 Appendix Survey and interview questionnaires. (PDF)

S2 Checklist Good reporting of a mixed methods study. (PDF)

S3 Table Participant characteristics. (PDF)

S4 Table Supports used/received by IMGs since being in Australia. (PDF)

S5 Table Usefulness of proposed supports for IMGs. (PDF)

S6 Table Summary of IMG-led recommendations. (PDF)

S7 Fig Responsibilities of stakeholders in IMG support. (PDF)
