# Supplementary material for: Supporting international medical graduates–what can be done better? A sequential explanatory mixed-methods study
Source: PLoS One. 2025 Aug 19;20(8):e0330558. doi: 10.1371/journal.pone.0330558 (PMC12364341; doi:10.1371/journal.pone.0330558)
Supplement: S1 Appendix — (PDF) [file pone.0330558.s001.pdf]

SURVEY QUESTIONNAIRE

This final section is about supports which may be offered to help IMGs in Australia.

**I am satisfied with current support from my workplace**<br><br>*(Single answer response)*	○ Strongly agree<br>○ Agree<br>○ Neutral<br>○ Disagree<br>○ Strongly disagree
**I am satisfied with current support from other institutions e.g., colleges**<br><br>*(Single answer response)*	○ Strongly agree<br>○ Agree<br>○ Neutral<br>○ Disagree<br>○ Strongly disagree

Below are some suggested supports for IMGs. How useful would such supports be, IF offered to IMGs working in Australia?
(Single answer response per item)

	Not useful at all	Marginally useful	Moderately useful	Very useful
Streamlining bureaucratic processes between institutions, e.g., immigration, workplaces, specialty colleges, registration and assessment boards	○	○	○	○
Modifying assessment requirements, based on recognition of previous qualifications/experience	○	○	○	○
Recognition and matching of previous qualifications/experience to future allocated jobs	○	○	○	○
Individualised career planning	○	○	○	○
Communication and language support	○	○	○	○
Cultural competency induction courses, e.g., Australia's values, systems, laws, indigenous health, LGBTQI+ health	○	○	○	○
Established departments to provide ongoing support to IMGs, including monitoring IMG rights	○	○	○	○
Established mentoring and peer support systems	○	○	○	○
Anonymisation of names on applications or complaints processes	○	○	○	○
Facilitation of mandatory requirements e.g., by accrediting bridging courses or work placements	○	○	○	○

Fostering a socially inclusive environment in the workplace	○	○	○	○
Consulting IMGs in qualification recognition, training and assessment of future programs	○	○	○	○

**Of the above supports, which have you personally used or received since being in Australia?**<br><br>**Please select all that apply**<br><br>*(Multiple response options available)*	☐ Streamlining bureaucratic processes between institutions, eg, immigration, workplaces, specialty colleges, registration and assessment boards<br><br>☐ Modifying assessment requirements, based on recognition of previous qualifications/experience<br><br>☐ Recognition and matching of previous qualifications/experience to future allocated jobs<br><br>☐ Individualised career planning<br><br>☐ Communication and language support<br><br>☐ Cultural competency induction courses e.g., Australia's values, systems, laws, indigenous health, LGBTQI+ health<br><br>☐ Established department to provide ongoing support to IMGs, including monitoring IMGs rights<br><br>☐ Established mentoring and peer support systems<br><br>☐ Anonymisation of names on applications or complains processes<br><br>☐ Facilitation of mandatory requirements e.g., by accrediting bridging courses or work placements<br><br>☐ Socially inclusive environment in the workplace<br><br>☐ Consultation of IMGs in qualification recognition, training and assessment of future programs

QUALITATIVE INTERVIEW GUIDE QUESTION

Please share any ideas you have for bettering IMG experiences in Australia (probe for: institutional: mentoring, peer groups, orientation, colleges, licencing/exams, workplace systems; workplace culture, interpersonal relations etc; advice for IMGs)
