# Supplementary material for: Supporting international medical graduates–what can be done better? A sequential explanatory mixed-methods study
Source: PLoS One. 2025 Aug 19;20(8):e0330558. doi: 10.1371/journal.pone.0330558 (PMC12364341; doi:10.1371/journal.pone.0330558)
Supplement: S2 Checklist — (PDF) [file pone.0330558.s002.pdf]

Good Reporting of a Mixed Methods Study (GRAMMS) checklist

(O'Cathain A, Murphy E, Nicholl J. The quality of mixed methods studies in health services research. J Health Serv Res Policy. 2008;13: 92-98.)

Guideline	Relevant section in article
1. Describe the justification for using a mixed methods approach to the research question	Methods - study design and setting
2. Describe the design in terms of the purpose, priority and sequence of methods	Methods- study design and setting
3. Describe each method in terms of sampling, data collection and analysis	Methods- participants, recruitment and sample size; instrument design and data collection<br>Analysis and reporting
4. Describe where integration has occurred, how it has occurred and who has participated in it	Analysis and reporting<br>Results: Triangulation of findings
5. Describe any limitation of one method associated with the present of the other method	Limitations of study
6. Describe any insights gained from mixing or integrating methods	Results: Triangulation of findings<br>Discussion: First paragraph; Strengths and Limitations of study
