# Supplementary material for: Supporting international medical graduates–what can be done better? A sequential explanatory mixed-methods study
Source: PLoS One. 2025 Aug 19;20(8):e0330558. doi: 10.1371/journal.pone.0330558 (PMC12364341; doi:10.1371/journal.pone.0330558)
Supplement: S3 Table — (PDF) [file pone.0330558.s003.pdf]

**Participant characteristics**

| | | *Survey participants* | *Interview participants* |
|---|---|---|---|
| **Age** | Range of years (mean) | 24-78 (40.4) | 27-60 (40.7) |
| | | *Number of participants (%)* | *Number of participants (%)* |
| **Gender** | Male | 71/252 (28.2%) | 9/36 (25%) |
| | Female | 179/252 (71%) | 27/36 (75%) |
| | Other | 2/252 (0.8%) | 0 |
| | ***Total*** | ***252*** | ***36*** |
| **Ethnicity** | Indian | 68/243 (28%) | No data |
| | Asian | 67/243 (27.6%) | |
| | British/Irish | 28/243 (11.5%) | |
| | European | 23/243 (9.5%) | |
| | Middle Eastern | 20/243 (8.2%) | |
| | Hispanic/Latino | 9/243 (3.7%) | |
| | African/ Caribbean | 8/243 (3.3%) | |
| | Eastern European | 4/243 (1.6%) | |
| | Polynesian | 1/243 (0.4%) | |
| | Mixed heritage | 10/243 (4.1%) | |
| | Prefer not to say/ other | 5/243 (2.1%) | |
| | ***Total*** | ***243*** | |
| *TRAINING OUTSIDE AUSTRALIA* | | | |
| **Most common country of primary medical qualification (PMQ)** | | 1. India: 45/230 (19.6%)<br>2. UK: 33/230 (14.4%)<br>3. China: 15/230 (6.5%<br>4. Ireland: 14/230 (6.1%)<br>5. Others: 123/230 (53.5%) | 1. India: 9/36 (25%)<br>2. UK: 7/36 (19.4%)<br>3. Egypt: 3/36 (8.3%)<br>4. South Africa: 3/36 (8.3%)<br>5. Others: 14/36 (38.9%) |

| | | | |
|---|---|---|---|
| **Completed or partially completed post-graduate training overseas** | yes | 138/236 (58.5%) | 23/36 (63.9%) |
| | no | 98/236 (41.5%) | 13/36 (36.1%) |
| | ***Total*** | ***236*** | ***36*** |
| **Current clinical employment level in Australia** | Consultant (GP or Specialist) | 74/190 (39%) | 13/36 (36.1%) |
| | Registrar or Fellow or CMO | 55/190 (29%) | 13/36 (36.1%) |
| | Resident Medical Officer or Intern | 52/190 (27.4%) | 9/36 (25%) |
| | Other/ prefer not to say | 9/190 (4.7%) | 1/36 (2.8%) (unemployed) |
| | ***Total*** | ***190*** | ***36*** |
| **Non-clinical jobs** | Education | 9/24 (54.1%) | 2/36 (5.6%) |
| | Research | 4/24 (16.7%) | |
| | Medical Administration | 4/24 (16.7%) | |
| | Others | 7/24 (29.2%) | |
| | ***Total*** | ***24*** | |
