# Supplementary material for: Supporting international medical graduates–what can be done better? A sequential explanatory mixed-methods study
Source: PLoS One. 2025 Aug 19;20(8):e0330558. doi: 10.1371/journal.pone.0330558 (PMC12364341; doi:10.1371/journal.pone.0330558)
Supplement: S4 Table — (PDF) [file pone.0330558.s004.pdf]

# Supports used/received by IMGs since being in Australia

| *"Of the above supports, which have you personally used or received since being in Australia?"* | | | |
|---|---|---|---|
| **Ranking** | **Support received/used** | **N (/134)** | **percentage** |
| 1 | Cultural competency induction courses eg, Australia's values, systems, laws, indigenous health, LGBTQI + health | 46/134 | 34.3% |
| 2 | Socially inclusive environment in the workplace | 40/134 | 29.9% |
| 3 | Established mentoring and peer support systems | 31/134 | 23.1% |
| 4 | Recognition and matching of previous qualifications/ experience to future allocated jobs | 29/134 | 21.6% |
| 5 | Modifying assessment requirements, based on recognition of previous qualifications/experience | 26/134 | 19.4% |
| 6 | Streamlining bureaucratic processes between institutions e.g., immigration, workplaces, specialty colleges, registration and assessment boards | 21/134 | 15.7% |
| 7 | Individualised career planning | 20/134 | 14.9% |
| 8 | Established department to provide ongoing support to IMGs, including monitoring IMGs rights | 17/134 | 12.7% |
| 9 | Communication and language support | 16/134 | 11.9% |
| 10 (equal ranking) | Consultation of IMGs in qualification recognition, training and assessment of future programs | 14/134 | 10.4% |
| | Facilitation of mandatory requirements e.g., by accrediting bridging courses or work placements | 14/134 | 10.4% |
| 12 | Anonymisation of names on applications or complaints processes | 6/134 | 4.5% |
