# Supplementary material for: Supporting international medical graduates–what can be done better? A sequential explanatory mixed-methods study
Source: PLoS One. 2025 Aug 19;20(8):e0330558. doi: 10.1371/journal.pone.0330558 (PMC12364341; doi:10.1371/journal.pone.0330558)
Supplement: S5 Table — (PDF) [file pone.0330558.s005.pdf]

Usefulness of proposed supports for IMGs

"How useful would such supports be, if offered to IMGs working in Australia?"					
	Not useful at all	Marginally useful	Moderately useful	Very useful	n
Streamlining bureaucratic processes between institutions, e.g., immigration, workplaces, specialty colleges, registration and assessment boards	5 (2.7%)	20 (10.6%)	38 (20.1%)	126 (66.7%)	189
Modifying assessment requirements, based on recognition of previous qualifications/ experience	7 (3.7%)	18 (9.4%)	42 (21.9%)	125 (65.1%)	192
Recognition and matching of previous qualifications/ experience to future allocated jobs	8 (4.2%)	19 (10%)	45 (23.7%)	118 (62.1%)	190
Individualized career planning	5 (2.6%)	23 (12.1%)	60 (31.6%)	102 (53.7%)	190
Communication and language support	17 (9.0%)	29 (15.3%)	66 (34.9%)	77 (40.7%)	189
Cultural competency induction courses, eg, Australia's values, systems, laws, indigenous health, LGBTQI+ health	9 (4.8%)	30 (16.0%)	69 (36.9%)	79 (42.3%)	187
Established departments to provide ongoing support to IMGs, including monitoring IMG rights	7 (3.7%)	22 (11.7%)	47 (25%)	112 (59.6%)	188
Established mentoring and peer support systems	5 (2.7%)	21 (11.2%)	50 (26.7%)	111 (59.4%)	187
Anonymization of names on applications	11 (5.8%)	34 (17.9%)	48 (25.3%)	97 (51.1%)	190

or complaints processes					
Facilitation of mandatory requirements eg, by accrediting bridging courses or work placements	8 (4.2%)	27 (14.3%)	59 (31.2%)	95 (50.3%)	189
Fostering a socially inclusive environment in the workplace	5 (2.7%)	18 (9.5%)	64 (33.9%)	102 (54.0%)	189
Consulting IMGs in qualification recognition, training and assessment of future programs	4 (2.1%)	16 (8.5%)	51 (27.1%)	117 (62.2%)	188
