# Supplementary material for: Supporting international medical graduates–what can be done better? A sequential explanatory mixed-methods study
Source: PLoS One. 2025 Aug 19;20(8):e0330558. doi: 10.1371/journal.pone.0330558 (PMC12364341; doi:10.1371/journal.pone.0330558)
Supplement: S6 Table — (PDF) [file pone.0330558.s006.pdf]

Summary of IMG-led recommendations

Advice for Institutions (AHPRA, AMC, specialty colleges, government bodies etc) and workplaces	
Improve operation of bureaucratic systems	• centralise, streamline and coordinate services<br>• improve information sharing including transparency of costs and career options<br>• reduce time and financial costs
Support, integrate, develop and mobilise IMGs	• review current registration and assessment policies<br>• revise exam and feedback processes. Focus on clinical assessment (e.g., WBA or similar)<br>• accredit observerships and bridging courses<br>• case-by-case recognition of prior experience or good standing<br>• create overarching advocacy body and access to liaison officers<br>• facilitate connectivity and experience sharing (e.g. mentoring, peer support groups)<br>• provide IMG-specific educational sessions with follow-up<br>• prioritise educational support in non-metropolitan locations<br>• deliver constructive feedback
Reduce discrimination and risk of exploitation	• review immigration policies and abolish visa links to employers<br>• IMG representation and inclusion in policies<br>• deliver fairer work conditions<br>• nurture trainees, especially in non-metropolitan locations<br>• promote cultural safety and harmony in the workplace<br>• encourage IMGs to share their stories and showcase their skills
**Advice for IMG peers**	
Attitude and adaptation	• be proactive, positive and resilient<br>• anticipate some challenge during the adjustment phase<br>• harness your agency and adapt to circumstances<br>• self-reflect<br>• learn to integrate with other staff and locals<br>• be confident. Speak up and give your opinion.<br>• ask for help<br>• rise above racism
Be prepared and seek advice early	• arm yourself with knowledge<br>• identify your deficits and seek ways to upskill accordingly<br>• seek honest feedback<br>• know your rights; be brave and stand up for yourself<br>• actively seek out support and mentors
Consider life stages	• plan according to your life-stages and priorities<br>• be mindful of potential competing distractions
