# Supplementary figures and images for: Supporting international medical graduates–what can be done better? A sequential explanatory mixed-methods study
Source: PLoS One. 2025 Aug 19;20(8):e0330558. doi: 10.1371/journal.pone.0330558 (PMC12364341; doi:10.1371/journal.pone.0330558)

## Responsibilities of stakeholders in IMG support

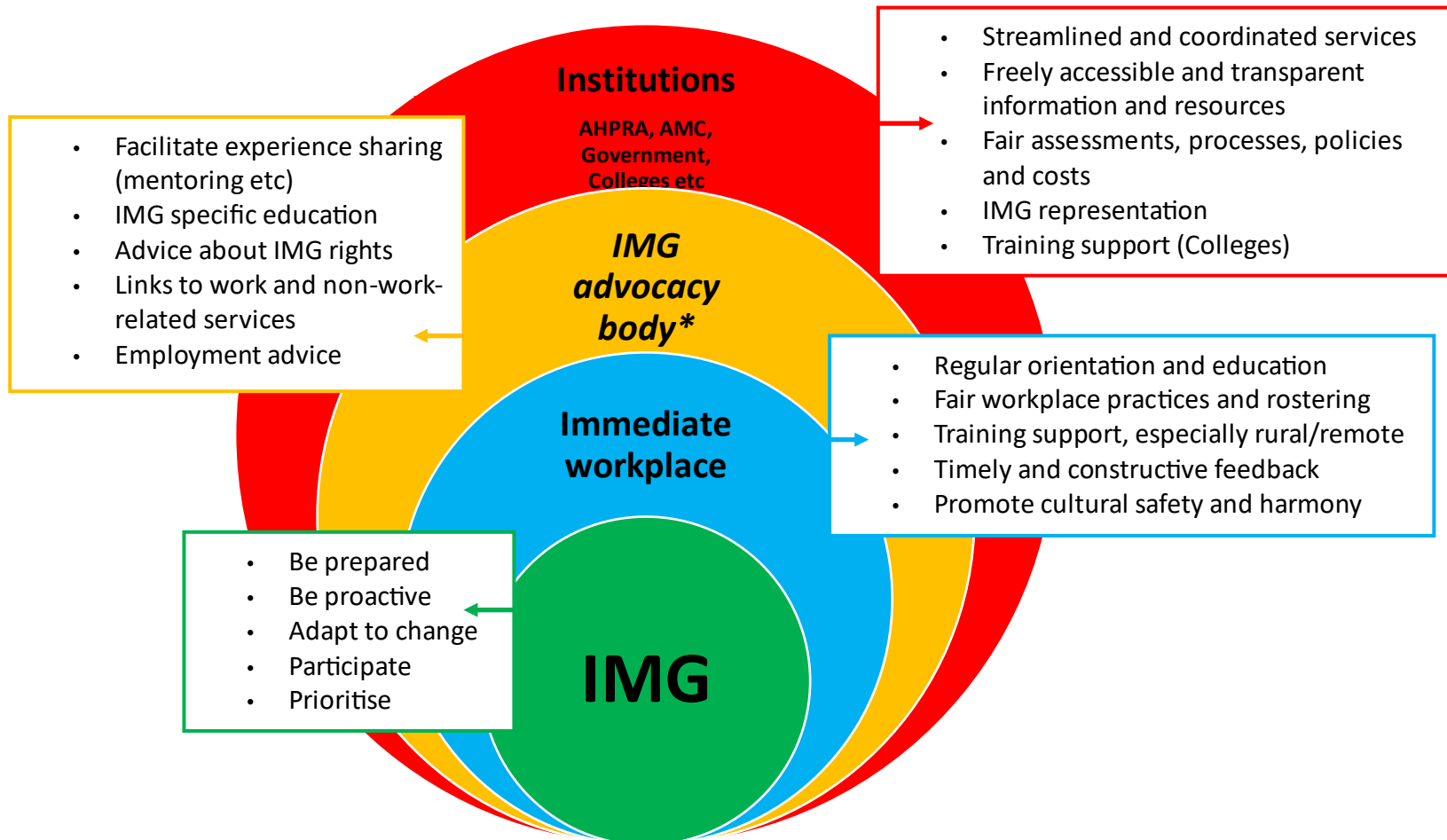

*\*Proposed new stakeholder service*

Supplement: S7 Fig — (PDF) [file pone.0330558.s007.pdf]
